# The Influence Mechanism of Interaction Quality in Live Streaming Shopping on Consumers’ Impulsive Purchase Intention

**DOI:** 10.3389/fpsyg.2022.918196

**Published:** 2022-07-07

**Authors:** Guangming Li, Yue Jiang, Liting Chang

**Affiliations:** Business School, Hohai University, Nanjing, China

**Keywords:** live streaming shopping, interaction quality, emotion, impulsive purchase intention, extroverted personality

## Abstract

As an emerging mode of online shopping, live streaming shopping has the characteristics of high interactivity. Live streaming shopping has achieved many positive results, but it leads to the phenomenon of consumers’ impulsive purchase. To analyze the reasons for impulsive purchase intention, we can make up for the lack of attention paid to this issue. Therefore, this study explores the influence mechanism of interaction quality between anchors and consumers on consumers’ emotion and impulsive purchase intention from the perspective of interaction quality combined with cognitive evaluation theory. A total 407 samples were collected by a questionnaire survey to test the research model. The results showed that: interaction quality (responsiveness, professionalism, informativeness, and personalization) had a significantly positive impact on consumers’ emotion; consumers’ emotion played a mediating role between interaction quality and impulsive purchase intention; consumers’ extroverted personality significantly negatively moderated the relationship between professionalism, informativeness and consumers’ emotion. This study can provide theoretical basis and reference for enterprises and anchors to carry out marketing activities and consumers’ rational consumption.

## Introduction

Since the rise of live streaming shopping mode in 2016, it has developed very rapidly and is still very popular today. According to the China Internet Network Information Center [CNNIC] (2022), as of December 2021, the number of e-commerce live broadcast users in China was 464 million, an increase of 75.79 million compared with December 2020, accounting for 44.9% of the total netizens^1^. Webcast has become the favorite shopping method of consumers, and more than 90% of e-commerce live users have ever purchased live products^2^. On the pre-sale day of “Double 11” only in 2021, the transaction volume of Taobao live broadcast exceeded 53 billion CNY (7.79 billion United States dollars), exceeding 12% of the live broadcast e-commerce market in 2019. At the same time, more and more stars, entrepreneurs, other well-known persons and government officials have joined the live broadcast. At present, live streaming shopping has become an important force to upgrade consumption, help rural revitalization and promote national economic development. However, with its rapid development, live streaming shopping has also triggered a common phenomenon of impulsive purchase among consumers. According to the report of the China Consumers Association, 44.1% of the respondents indicated that impulsive purchase phenomenon in live streaming shopping was serious^3^. The return problems caused by impulsive purchase not only increase the transaction cost of consumers, but waste the resources of enterprises. Therefore, it is necessary to explore the influence factors and mechanism of consumers’ impulsive purchase intention in live streaming shopping.

According to existing research, the influencing factors of online impulsive purchase can be divided into product factors, consumer factors and environment factors. Product factors are mainly studied from the perspective of product type (Liao et al., 2016). Consumer factors include cognition (Chen et al., 2016; Lee and Chen, 2021), emotion (Ozer and Gultekin, 2015), personality traits (Iyer et al., 2019) and so on. In the traditional online shopping environment, environment factors mainly include website characteristics (Liu et al., 2017), website promotion and word-of-mouth (Lo et al., 2016), online reviews (Huang, 2016), network interaction (Jiang et al., 2014) and so on. These studies provide some reference for this study to explore the influencing factors of impulsive purchase in live streaming shopping. However, compared with the traditional e-commerce shopping mode, live streaming shopping is more interactive (Zuo and Xiao, 2021). Anchors can interact with consumers in real time through words, expressions and body language, with richer interactive ways and deeper interactive content. Therefore, the skills and interaction quality of different anchors are very different, and they can bring completely different live streaming shopping experience to consumers. Based on the theory of impulsive purchase behavior from the emotional perspective (Chang et al., 2012), consumers will have a strong emotional response to the stimulus in the shopping situation, which leads them to make impulsive purchase without rational thinking. It can be seen that the difference in the interaction quality between anchors and consumers may bring different emotional experience to consumers, and then affect their impulsive purchase intention. Therefore, based on the cognitive evaluation theory, the authors explore the influence mechanism of interaction quality on consumers impulsive purchase intention, and make an empirical test. The research conclusions have practical significance for guiding the sustainability development of related enterprises and rational purchasing behavior of consumers. At the same time, it also helps to enrich research in the field of live streaming shopping and impulsive purchase.

## Literature Review and Research Hypotheses

### Interaction Quality

In the field of traditional offline service, interaction quality refers to the feelings of consumers and service personnel in the process of contact. Among them, the attitude, behaviors and skills of service personnel are important criteria for measuring the interaction quality (Brady and Cronin, 2001). The subjects of interaction are consumers and service personnel, and their interaction is mainly through face-to-face contact. Different from the interaction of the physical scene, the interaction of live streaming shopping scene relies on the network technology. It can realize the real-time interaction of multiple subjects (Liu et al., 2020), including the interaction between consumers and anchors, the other consumers. Anchors are the interaction core of live streaming shopping (Li M. W. et al., 2021). Therefore, this paper mainly studies the interaction quality between anchors and consumers. It refers to consumers’ cognitive evaluation on the interaction effect between them and anchors in the process of live streaming shopping.

Scholars put forward that the dimensions of interaction quality mainly include professionalism and personalization in offline service environment (Winsted, 1997; Brady and Cronin, 2001). However, it may have some new dimensions in the context of live streaming shopping. On the one hand, professional anchors are good at creating a good interactive atmosphere for consumers, which can improve consumers’ pleasure, and then satisfy their emotional needs (Hou et al., 2020; Liu et al., 2020). Professional anchors use some original content and forms to display products, and they can use natural and vivid language to promote consumers to communicate in interaction actively (Zhang M. et al., 2021). What’s more, professional anchors have skilled products knowledge that they can screen out useful information from heavy product information for consumers (Lu and Chen, 2021). On the other hand, anchors can answer the questions raised by consumers according to the barrage information, and meet the individual needs of consumers (Hou et al., 2020). If anchors can provide personalized service to consumers during interaction, the emotional distance between them will be narrowed, and they can be connected more closely (Hou et al., 2020; Zhang M. et al., 2021). In addition, the interaction in the live streaming shopping situation is intuitive and immediate (Liu et al., 2020). On the one hand, live streaming shopping provides real-time exhibits display and sales scenes, which can’t be edited and modified later (Chen et al., 2019; Zhang M. et al., 2021). In the process of live broadcast, anchors can provide complete and accurate information, which greatly guarantees the completeness of information (Sun et al., 2019; Zhang M. et al., 2021). In the traditional online shopping platform, consumers can only view product information through the text, pictures and videos uploaded by merchants. However, live streaming shopping adopts the way of real-time video transmission, so that consumers can observe product details and view promotional information in all directions (Chen et al., 2019). At the same time, consumers can get more complete information through other consumers’ concerns in the barrage (Hou et al., 2020; Wongkitrungrueng et al., 2020). Therefore, consumers can get richer and more effective product information in the interaction process. On the other hand, live streaming shopping is based on modern information technology, which can break the limitation of time and space and provide a platform for real-time communication, so that consumers can interact with anchors in real time. Through real-time interaction, consumers can quickly get feedback from anchors (Zhang S. et al., 2021). To sum up, this paper divides the interaction quality of live streaming shopping into four dimensions: professionalism, personalization, informativeness and responsiveness. Professionalism refers to the degree to which anchors can provide consumers with correct, effective product knowledge or experience and good communication skills (Brady and Cronin, 2001). Personalization refers to the degree to which anchors can provide targeted service to consumers (Winsted, 1997). Informativeness refers to the degree that anchors can provide consumers with information related and complete to live broadcast products (Mathwick et al., 2008). Responsiveness refers to the frequency and speed that anchors can provide consumers with the required information (Jiang et al., 2014).

### Impulsive Purchase

Impulsive purchase usually means that consumers have no purchase plans or intention in advance, and they immediately implement the purchase behaviors only based on sudden or temporary thoughts (Chan et al., 2017; Iyer et al., 2019). Generally speaking, impulsive purchase is a kind of complex purchase behavior with a sudden, uncontrollable and hedonic thoughts (Iyer et al., 2019). Previous studies have proposed stimulus theory and emotion theory to explain the impulsive purchase mechanism. They almost emphasize the unilateral role of stimulus factors or emotional factors. However, the essence of impulsive purchase is that consumers form cognitive and emotional reactions in the purchase process (Chang et al., 2012). Under the influence of marketing incentives, consumers have positive emotional response. It is more difficult for them to restrain the desire to buy products which leads to impulsive purchase (Chang et al., 2012; Lin and Lo, 2016). In the live streaming shopping situation, consumers are stimulated by the interaction with anchors, and have an evaluation of the interaction. During the process, consumers get a good experience so that fluctuate positive emotion (Liu et al., 2018). When consumers are in a positive emotional state, they will pay more attention to products. Thus, they may overestimate their own economic ability and needs so as to increase the possibility of impulsive purchase (Khachatryan et al., 2018). Impulsive purchase intention is a kind of psychological desire, meantime is an important antecedent to determine impulsive purchase behavior (Chan et al., 2017). Therefore, this paper mainly studies impulsive purchase intention.

### Emotion

Emotion is an emotional state generated after cognitive evaluation of external stimuli (Bagozzi et al., 1999), which includes three dimensions: arousal, pleasure and dominant (Kapucu et al., 2018). However, scholars later found that the dominant dimension had little influence on consumer behavior in the empirical study, so the dominant dimension was seldom used (Russell, 1979; Thomas et al., 2003). Therefore, this paper adopts this research tradition to measure emotion from two dimensions: arousal and pleasure. Arousal refers to the degree to which people feel stimulated, active, or alert. It is the active state of the individual nervous system, which can be divided into high and low levels. High arousal is characterized by concentration, while low arousal is characterized by relaxation (Yang et al., 2021). Arousal is usually measured with terms such as stimulated/relaxed, excited/calm, and aroused/unaroused (Eroglu et al., 2003). In this research, arousal is defined as the degree to which individuals involved in live streaming shopping feel stimulated, active or excited during the process of watching. Pleasure refers to the degree to which people feel good, joyful, happy or satisfied in a certain situation (Michael et al., 2021). Pleasure is usually measured with terms such as happy/unhappy, pleased/annoyed, and contented/melancholic (Eroglu et al., 2003). Therefore, pleasure emotion in this study refers to the degree to which individuals feel good, happy, or satisfied in the live streaming shopping situation.

### Research Hypotheses

Based on cognitive evaluation theory, emotion does not originate from specific events or environment, but from people’s cognitive evaluation (Bagozzi et al., 1999). Therefore, interaction quality between consumers and anchors will affect the generation of consumers’ emotion.

The interaction quality between consumers and anchors will positively affect their arousal emotion. First, when anchors can answer consumers’ questions in time, communicate with them actively, and meet their needs, which can improve consumers’ attention, and make consumers participate in interaction actively (Herman et al., 2018). Meantime, the users’ arousal emotion level is improved (Lăzăroiu et al., 2020). Secondly, professional anchors are good at creating a relaxed and happy atmosphere for consumers, displaying products in a vivid way (Liu et al., 2020). It will make consumers more focused and generate cognitive evaluation during the process of watching live broadcasts, so that their arousal emotion are improved (Lee and Chen, 2021). In addition, professional purchasing advice will reduce the perceived risk of consumers (Lăzăroiu et al., 2020), which make consumers generate positive cognitive evaluation. Consumers attain pleasant purchasing experience, and then their attention are improved (Ha and Lennon, 2010). Thirdly, anchors provide consumers with comprehensive product information, meeting consumers’ information needs. Consumers become more interested and allocate more attention to the interaction with the host (Zhang M. et al., 2021). Meanwhile, the degree of consumers’ nervous system arousal is increased. Finally, during the live broadcast, anchors provide personalized service for consumers in order to shorten the distance with consumers (Xu and Ye, 2020; Lin et al., 2021). At the same time, they provide targeted purchase suggestions according to consumers’ needs. Consumers will attain the convenient experience of live streaming shopping, so they form positive cognitive evaluation and have a higher degree of arousal emotion (Khachatryan et al., 2018). Thus, in the situation of live streaming shopping, responsiveness, professionalism, informativeness and personalization will have an impact on consumers’ arousal emotion. Based on this, the following assumptions are put forward:

Hypothesis 1a (H1a): Responsiveness has a significant positive effect on consumers’ arousal emotion.

Hypothesis 1b (H1b): Professionalism has a significant positive effect on consumers’ arousal emotion.

Hypothesis 1c (H1c): Informativeness has a significant positive effect on consumers’ arousal emotion.

Hypothesis 1d (H1d): Personalization has a significant positive effect on consumers’ arousal emotion.

The interaction quality between consumers and anchors will positively affect their pleasure emotion. Firstly, anchors meet the needs of consumers in an instant response that will strengthen the interaction between consumers and anchors. Thus, consumers can participate in the interaction with a more enjoyable mentality so as to form a higher pleasure (Lin et al., 2021). Secondly, professional anchors can provide consumers with more effective product recommendations, reduce their confusion. It will enable them to form a positive perception of products (Lee and Chen, 2021), and then generate good emotion (Xu et al., 2019). In addition, professional anchors can create a cheerful and relaxed atmosphere, making consumers feel happy, comfortable and satisfied in the process of interaction (Lee and Chen, 2021). Thirdly, anchors can effectively answer the questions raised by consumers, meet their information needs. They will reduce the time cost and energy that consumers need to pay to collect information. After that, consumers can form positive cognitive evaluation and improve their pleasure (Zhang S. et al., 2021). Finally, some studies have proved that shopping platform provides personalized products and service, which is helpful for consumers to have a pleasure emotion (Liu et al., 2018; Riegger et al., 2021). When consumers’ personalized needs are met, they will feel close to anchors and get a good shopping experience (Xu and Ye, 2020). Consumers’ positive evaluation of interaction makes them more pleasure. Thus, in the situation of live streaming shopping, responsiveness, professionalism, informativeness and personalization will have an impact on consumers’ pleasure emotion.

Hypothesis 2a (H2a): Responsiveness has a significant positive effect on consumers’ pleasure emotion.

Hypothesis 2b (H2b): Professionalism has a significant positive effect on consumers’ pleasure emotion.

Hypothesis 2c (H2c): Informativeness has a significant positive effect on consumers’ pleasure emotion.

Hypothesis 2d (H2d): Personalization has a significant positive effect on consumers’ pleasure emotion.

Emotion is an emotional state generated after cognitive evaluation of external stimuli, may lead to some certain behaviors (Bagozzi et al., 1999). ABC effect hierarchy model points out consumers’ cognition will affect emotion, and then affect consumers’ behavior. This reveals that emotion mediate between cognition and behavior (Solomon, 2018). In the process of live streaming shopping, consumers evaluate the interaction effect with anchors, and generate emotional reaction, then their behavior will have a series of changes. Specifically, on the one hand, consumers will evaluate the interaction effect with anchors, causing psychological wave motion and generating arousal emotion (Solomon, 2018; Liu et al., 2020). This kind of emotional reaction will not only increase consumers’ attention to live broadcast products, but also encourage consumers to form impulsive purchase in order to calm down their emotional fluctuations (Iyer et al., 2019; Zhang et al., 2020). That is, arousal emotion plays an intermediary role between interaction quality and impulsive purchase intention. On the other hand, consumers attain a positive response in the interaction with anchors, resulting in a good experience, and further forming positive cognitive evaluation, resulting in a pleasure emotion (Lee and Chen, 2021; Xu et al., 2021). This kind of positive emotional reaction will make it difficult for consumers to resist the temptation of products and overestimate their actual needs, thus making it easier to generate impulsive purchase intention (Zhang et al., 2020). That is, pleasure emotion plays an intermediary role between the interaction quality and impulsive purchase intention. The intermediary role in other online shopping situation has also been verified: consumers’ perception of the atmosphere of online shopping will affect consumers’ arousal and pleasure emotion, which will lead to impulsive purchase. Furthermore, the stronger consumers’ arousal and pleasure emotion is, the easier to have impulsive purchase (Koo and Ju, 2010; Liu et al., 2018). Accordingly, the following assumptions are put forward:

Hypothesis 3a (H3a): Arousal emotion plays an intermediary role between interaction quality and consumers’ impulsive purchase intention.

Hypothesis 3b (H3b): Pleasure emotion plays an intermediary role between interaction quality and consumers’ impulsive purchase intention.

According to cognitive evaluation theory, individual differences in personality lead to different ways of processing information (Bagozzi et al., 1999). Therefore, the process of cognitive evaluation will be affected by consumer personality traits. One of the most common frameworks used to describe personality traits is Big Five Personality (Barrick and Mount, 1991). Extroverted personality refers to the number and density of individual interpersonal interaction, the need for stimulation and the ability to obtain pleasure (Turban et al., 2016). The arousal theory points out that the arousal level of the cerebral cortex of extroverts is inherently low, and it is not easy to be activated or excited. Thus, they need to obtain more stimuli from the outside world to achieve a satisfactory level of arousal (Eysenck, 1994). In the process of live streaming shopping interaction, the higher level of extroverted personality of consumers, the less easily the cerebral cortex is awakened, and the less excited it is. To achieve the same level of arousal, there is a greater need to receive stimuli. Therefore, it is more difficult for anchors to meet consumers’ demand for stimulation in interaction. At the same level of stimulation, consumers with high level of extroverted personality will have less pleasure and arousal emotion. Based on this, the following assumptions are put forward:

Hypothesis 4a (H4a): Extroverted personality negatively regulates the influence of responsiveness on arousal emotion.

Hypothesis 4b (H4b): Extroverted personality negatively regulates the influence of professionalism on arousal emotion.

Hypothesis 4c (H4c): Extroverted personality negatively regulates the influence of informativeness on arousal emotion.

Hypothesis 4d (H4d): Extroverted personality negatively regulates the influence of personalization on arousal emotion.

Hypothesis 4e (H4e): Extroverted personality negatively regulates the influence of responsiveness on pleasure emotion.

Hypothesis 4f (H4f): Extroverted personality negatively regulates the influence of professionalism on pleasure emotion.

Hypothesis 4g (H4g): Extroverted personality negatively regulates the influence of informativeness on pleasure emotion.

Hypothesis 4h (H4h): Extroverted personality negatively regulates the influence of personalization on pleasure emotion.

**FIGURE 1:**
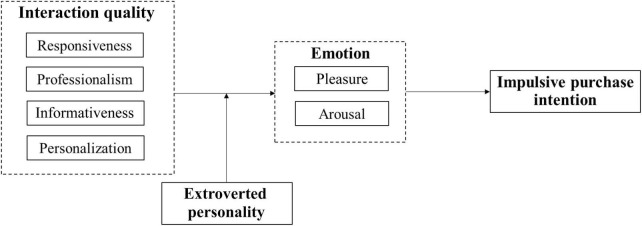
Interaction quality.

The research model of this paper is constructed as shown in Figure 1.

## Methodology

### Questionnaire Design and Variable Measurement

The questionnaire consists of three parts. Firstly, the basic situation of the respondents who watch and purchase in the live broadcast room. Among them, “Have you watched live streaming shopping?” and “Have you ever purchased in the broadcast room?” are the screening items. Samples with no purchase experience in the live broadcast room will be excluded as invalid questionnaires. The second part is the details of the investigator’s purchase experience in the live broadcast room. By setting questions, consumers can recall their most recent purchase experience. Meantime, guide them to associate the interaction and purchase with anchors in the live broadcast room. Then, measure with using a scale. The third part is some basic information of the investigators, including gender, age, education level, income, and weekly watching live broadcast time. In addition, this paper has designed the 3rd, 6th, 9th, and 12th items of measuring extraversion personality as antisense question items (see Table 1). In order to ensure good reliability and validity of variable measurement, all variables measured in this paper refer to the mature scale in existing studies. Meanwhile, according to the specific situation of live streaming shopping and the feedback of respondents, the expressions of the measurement items are appropriately modified. All scale items adopt Likert 7-point scale (1 means totally disagree, 7 means totally agree, and the larger the number, the greater the degree of agreement). The sources of the scale are shown in Table 1. This study takes the gender, age, education level, monthly income level and the weekly watching live broadcast time as control variables.

**TABLE 1:** Variablew measurement items (*N* = 407).

Measurement variable	Measurement dimension	Measurement item	Document
Interaction quality	Responsiveness	When shopping in the live room, the anchor is willing and patient to answer my questions When shopping in the live broadcast room, the anchor quickly responds to my product consultation questions When shopping in the live room, the anchor’s response is closely related to the questions I mentioned	Jiang et al. (2014)
	Professionalism	I think the anchor I watch has professional product knowledge I think the anchor I watch has special communication skills I think the anchor I watch is good at mobilizing and creating the atmosphere of the live broadcast room	Brady and Cronin (2001)
	Informativeness	I think that through the introduction of the anchor, I have deepened my impression of the goods I think that through the introduction of the anchor, I have deepened my understanding of commodities I think that through the introduction of the anchor, I have get valuable information	Mathwick et al. (2008)
	Personalization	When shopping in the live room, I think the anchor can provide services according to my requirements When shopping in the live room, the anchor can put forward some suggestions on the purchase of goods according to my situation When shopping in the live room, I feel that I have got a targeted solution	Winsted (1997)
Emotion	Arousal emotion	When shopping in the live room, I am excited when browsing or buying When shopping in the live room, I am excited when browsing and buying When shopping in the live room, I am surprised when browsing and buying	Koo and Ju (2010)
	Pleasure emotion	When shopping in the live room, I feel happy when browsing or buying When shopping in the live room, I am satisfied when browsing or buying When shopping in the live room, I feel happy when browsing and buying	
Impulsive purchase intention		As soon as I saw the product recommended by the anchor live broadcast, I thought it was what I wanted When watching the live broadcast, the products recommended by the anchor will make me have a sudden and strong impulse to buy, even if the products are not in my purchase list I had no plan at all in advance, and I decided to buy it after seeing the anchor’s recommendation When I enter the live broadcast room, I want to spend money	Lee and Chen (2021)
Extroverted personality		I like to have many friends around me I laugh easily I don’t think I am particularly carefree^R^ I really like talking to people I like to stay where there are activities I often like to do things alone^R^ I often feel like I am full of energy I am a happy and passionate person I am not an optimist^R^ My life is very fast I am a very active person I’d rather work alone than lead others together^R^	Costa and Mccrae (1992)

*R, Represent Antisense items.*

### Pre-investigation

In order to ensure the validity and reliability, a small pre-survey was first conducted before the formal survey, and a total of 110 valid questionnaires were collected. SPSS 24.0 software was used for reliability and exploratory factor analysis. After deleting items with cross loadings and factor loadings less than 0.500, a total of 8 factors were rotated out. After revision, this scale retained 34 items and 8 factors, with a total explained variance of 79.135%. The Cronbach’s α coefficients of responsiveness, professionalism, informativeness, personalization, pleasure, arousal, impulsive purchase intention, and extraversion personality variables were 0.858, 0.860, 0.865, 0.862, 0.854, 0.894, 0.820, and 0.661. It could be seen that Cronbach’s α coefficients of eight variables were all above 0.60, which indicated that the scale had good reliability and could be used for formal investigation.

### Formal Investigation

In the formal investigation process, this study collected questionnaire data online by convenience sampling method. The authors distributed questionnaires to the circle of friends, Douban discussion group and other platforms from September to December 2021. A total of 500 questionnaires were collected. After excluding invalid questionnaires, there were 407 valid questionnaires, with an effective rate of 81.4%. The survey respondents were mainly people who had watched live streaming shopping, covering different provinces, autonomous regions and municipalities. Most of them were in East China, and less in Southwest and Northwest. The sample distribution in other provinces was balanced. Descriptive statistics of the samples were shown in Table 2. The sample characteristics were in line with the characteristics of young and highly educated users of China’s online shopping in the “2021 China Online Shopping Market Research Report” released by CNNIC in 2022.

**TABLE 2:** Descriptive statistics of the sample.

Characteristic variable	Option range	Percentage (%)	Characteristic variable	Option range	Percentage (%)
Gender	Man	45.6	Region	Eastern China	26.6
	Woman	54.4		Southern China	16.7
Age	Under 18 years old	0.2		Central China	14.8
	18–25 years old	29.7		North China	13.5
	26–30 years old	31.9		Northwest China	5.5
	30–40 years old	23.8		Southwest China	7.1
	40–50 years old	9.8		Northeast China	15.8
	50–60 years old	3.7	Monthly Income	Less than 2,000 yuan	14.3
	Over 60 years old	0.9		2,000–5,000 yuan	41.5
Education level	Junior high school and below	8.1		501–8,000 yuan	25.8
	Senior high school	10.2		801–11,000 yuan	14.3
	College for professional training	21.6		More than 1,000 yuan	4.1
	Undergraduate course	40.0	Watch live broadcast time per week	0–4 h	42.5
	Master degree or above	20.1		4–8 h	22.4
				8–12 h	19.7
				12–16 h	11.8
				More than 16 h	3.6

## Results

### Data Analysis

#### Common Method Deviation Test

Common Method Variance (CMV) is the overlap of variations among variables due to the same data sources, measuring tools and environment, which does not represent the real relationship between constructs. In order to minimize the influence of common method variance, this study adopted anonymous filling, reverse item design, and tried to match the language expression of consumers when designing the questionnaire. In addition, Harman single factor test was used to test whether there was common method variance after questionnaires were collected. The results of untwisted factor analysis showed that the variance explanatory quantity of the first factor was 41.160%, which was less than the critical value of 50% (Podsakoff and Organ, 1986), indicating that there was no common method variance in this study.

#### Reliability and Validity Test

This paper used AMOS 21.0 to carry out confirmatory factor analysis on formal sample data. The results showed that χ^2^/df = 1.767 < 5.000; RMSEA = 0.043 < 0.080; GFI = 0.887 > 0.850; CFI = 0.958; TLI = 0.952; IFI = 0.958 and NFI = 0.908, all greater than 0.900. Each fitting index was better than the fitting reference standard, which indicated that the model fitted well. The standardized coefficient of factor load between measured items and factors was between 0.622 and 0.902, which showed that the corresponding relationship between each measured item and factor was correct. The above data showed that the measurement model had reached an acceptable level.

According to the results of confirmatory factor analysis, the average variance extraction (AVE) and the combined reliability (CR) of each variable were calculated. The results were shown in Table 3: the CR was greater than 0.7, indicating that each variable had good reliability; the AVE of all variables was greater than 0.5, indicating that each variable had good convergent validity, and the square root of each variable AVE was greater than the correlation coefficient between them, indicating that each variable had good discriminant validity.

**TABLE 3:** Discriminant validity analysis of measurement model.

Variable	ME	SD	CR	AVE	1	2	3	4	5	6	7	8
1. XYX	5.165	1.229	0.862	0.675	**0.822**							
2. ZYX	5.148	1.128	0.813	0.592	0.528**	**0.769**						
3. XXX	5.267	1.224	0.860	0.673	0.557**	0.652**	**0.820**					
4. GXH	5.094	1.224	0.866	0.683	0.603**	0.633**	0.542**	**0.826**				
5. YY	5.193	1.320	0.890	0.730	0.544**	0.587**	0.589**	0.583**	**0.854**			
6. HX	4.955	1.348	0.895	0.740	0.470**	0.561**	0.491**	0.548**	0.758**	**0.860**		
7. CD	4.522	1.419	0.889	0.666	0.500**	0.495**	0.459**	0.442**	0.465**	0.450**	**0.815**	
8. WXX	4.440	0.978	0.922	0.522	0.383**	0.443**	0.465**	0.444**	0.460**	0.456**	0.425**	**0.722**

**p < 0.010 < 0.001, the same below. The diagonal data is the square root of the latent variable AVE value, and the font is bold. XYX, responsiveness; ZYX, professionalism; XXX, informativeness; GXH, personalization; YY, pleasure; HX, arousal; CD, impulsive purchase intention; WXX, extroverted personality.

### Hypothesis Test

#### The Main Effect Test

In this study, SPSS 24.0 was used for hierarchical regression analysis to test the research hypothesis, and the average value of measured items scores was used as the variable value. Before regression, this paper tested the variables for multicollinearity, and the results showed that the maximum value of VIF is 2.191, less than 10.0, which indicated that there was no multicollinearity problem and regression analysis could be carried out.

Specifically, Model 1 was a basic model with only control variables added to test its effect on arousal emotion. The results showed that gender, age, education level, monthly income and weekly watching live broadcast time did not affect the individual’s arousal emotion. Model 2 was based on model 1 with independent variables. At this time, the explanatory ability of the model for arousal emotion was improved by 38.400% (*f* = 63.405, *p* < 0.001). The results showed that responsiveness (β = 0.114, *p* < 0.050), professionalism (β = 0.271, *p* < 0.001), informativeness (β = 0.113, *p* < 0.050) and personalization (β = 0.244, *p* < 0.001) all had effects on arousal emotion. Therefore, it was assumed that 1a, 1b, 1c, and 1d were verified. Model 3 was a basic model with only control variables added to test its effect on pleasure emotion. The results showed that age and weekly watching live broadcast time negatively affected individual’s pleasure emotion. Model 4 was based on model 3 with independent variables. At this time, the explanatory ability of the model to pleasure emotion was improved by 44.400% (*f* = 88.711, *p* < 0.001). The results showed that responsiveness (β = 0.168, *p* < 0.001), professionalism (β = 0.212, *p* < 0.001), informativeness (β = 0.211, *p* < 0.001) and personalization (β = 0.219, *p* < 0.001) had positive effects on pleasure emotion. Therefore, it was assumed that 2a, 2b, 2c, and 2d were verified.

#### The Intermediary Test

For intermediary test, firstly, this study tested whether responsiveness, professionalism, informativeness and personalization of independent variables had significant effects on impulsive purchase intention of dependent variables. From Model 6, it could be seen that responsiveness, professionalism, informativeness, and personalization had significant effects on impulsive purchase intention (β = 0.298, *p* < 0.001; β = 0.232, *p* < 0.001; β = 0.157, *p* < 0.010; β = 0.126, *p* < 0.050). Secondly, this study tested whether the independent variables’ responsiveness, professionalism, informativeness and personalization had significant effects on the pleasure and arousal emotion of intermediary variables. It could be seen from models 2 and 4 that responsiveness, professionalism, informativeness and personalization had significant effects on the pleasure and arousal emotion. Finally, this study tested whether independent variables and intermediary variables had significant effects on dependent variables at the same time. It could be seen from models 7 and 8 that the effects of responsiveness, professionalism, informativeness and personalization on impulsive purchase intention were smaller than those in the first step (0.257 < 0.298; 0.172 < 0.232; 0.114 < 0.157; 0.116 < 0.126; 0.267 < 0.298; 0.175 < 0.232; 0.136 < 0.157; 0.121 < 0.126). The test results of intermediary effect were shown in Table 4. Therefore, arousal and pleasure emotion played a partial intermediary role in responsiveness, professionalism, informativeness, personalization and impulsive purchase intention. Therefore, it was assumed that 3a, 3b were verified.

**TABLE 4:** Main and intermediary effect test results.

Variable	Arousal emotion		Pleasure emotion		Impulsive purchase intention			
	M1	M2	M3	M4	M5	M6	M7	M8
Gender	0.004	−0.010	0.048	0.030	0.034	0.026	0.026	0.020
Age	−0.091	−0.036	−0.173***	−0.106**	0.005	0.034	0.041	0.050
Education level	−0.013	−0.028	−0.018	−0.028	−0.116*	−0.117**	−0.115**	−0.114*
Monthly income	0.057	0.031	0.017	−0.007	0.079	0.085	0.078	0.085
Watch the live broadcast every week	−0.082	−0.026	−0.144**	−0.078*	0.028	0.076	0.080	0.090
Responsiveness		0.114*		0.168***		0.298***	0.267***	0.257***
Professionalism		0.271***		0.212***		0.232***	0.175**	0.172**
Informativeness		0.113*		0.211***		0.157**	0.136*	0.114*
Personalization		0.244***		0.219***		0.126*	0.121*	0.116*
Pleasure								0.191***
Arousal							0.155**	
*R* ^2^	0.015	0.400	0.060	0.504	0.025	0.362	0.373	0.378
Δ*R*^2^	0.015	0.384	0.060	0.444	0.025	0.337	0.015	0.020
ΔF	1.031	63.405***	4.246***	88.711***	1.700	52.248***	9.600**	12.774***
Max (VIF)	1.268	2.191	1.268	2.191	1.268	2.191	2.046	2.107

*p < 0.050, **p < 0.010, and ***p < 0.001.

#### The Moderating Effect Test

For the moderating effect of extraversion personality, this paper used SPSS 24.0 to test by hierarchical regression method. Centering on responsiveness, professionalism, informedness, personalization and extraversion personality, the maximum VIF value was 2.246, indicating that there was no multicollinearity problem among the variables (see Table 5).

**TABLE 5:** The moderating effect of extraversion personality tests.

Variable	Arousal emotion					Pleasure emotion				
	M9	M10	M11	M12	M13	M14	M15	M16	M17	M18
Gender	−0.006	−0.007	−0.009	−0.008	−0.006	0.033	0.033	0.030	0.031	0.033
Age	−0.036	−0.036	−0.034	−0.037	−0.036	−0.106**	−0.106	−0.104**	−0.108**	−0.104**
Education level	−0.029	−0.028	−0.023	−0.024	−0.03	−0.029	−0.027	−0.021	−0.022	−0.026
Monthly income	0.029	0.038	0.046	0.046	0.028	−0.008	0.005	0.014	0.013	−0.003
Watch the live broadcast every week	−0.021	−0.019	−0.016	−0.017	−0.022	−0.074*	−0.072*	−0.068	−0.069	−0.071
Responsiveness	0.102*	0.092	0.090	0.088	0.103*	0.160***	0.146***	0.145**	0.143**	0.157***
Professionalism	0.247***	0.243***	0.234***	0.237***	0.247***	0.195***	0.189***	0.177***	0.182***	0.196***
Informativeness	0.071	0.060	0.050	0.042	0.073	0.180***	0.166***	0.154**	0.144**	0.171***
Personalization	0.209***	0.2028**	0.201***	0.186***	0.212***	0.194***	0.184***	0.184***	0.165***	0.185***
Extroverted	0.178***	0.176***	0.173***	0.162***	0.179***	0.129**	0.126***	0.123**	0.108**	0.127**
Responsiveness × extroverted		−0.055					−0.071			
Professionalism × extroverted			−0.094*					−0.121**		
Informativeness × extroverted				−0.127**					−0.159***	
Personalization × extroverted					−0.010					−0.035
*R* ^2^	0.423	0.425	0.429	0.433	0.423	0.516	0.520	0.526	0.532	0.517
Δ*R*^2^	0.407	0.002	0.006	0.010	0.000	0.456	0.004	0.010	0.016	0.001
ΔF	55.723***	1.489	4.271*	6.989**	0.043	74.482***	3.474	8.473**	13.252***	0.655
Max (VIF)	2.215	2.224	2.246	2.226	2.224	2.215	2.224	2.246	2.226	2.224

*p < 0.050, **p < 0.010, and ***p < 0.001.

Firstly, this study tested the role of extroverted personality in responsiveness, professionalism, informativeness, and personalization in influencing arousal emotion. Model 9 was only put into control variables, independent variables and adjustment variables, which accounted for 40.700% of arousal emotion variance (*f* = 55.723, *p* < 0.001). Model 10 was further put into responsiveness and extraversion personality, which had no significant change in the explanatory power of the variance of arousal emotion, and had no significant effect (β = −0.055, *p* > 0.050), indicating that extraversion personality failed to regulate the relationship between responsiveness and arousal emotion, H4a was not proved. In model 11, professionalism and extraversion personality were added on the basis of Model 9, and its explanatory power to the variance of arousal emotion was improved by 0.600% (*f* = 4.271, *p* < 0.050), and it had a significant negative influence (β = −0.094, *p* < 0.050), which indicated that extraversion personality negatively regulated the relationship between professionalism and arousal emotion, assuming H4b was proved. In model 12, informativeness and extroverted personality were added, and its explanatory power to arousal emotion variance was increased by 1.000% (*f* = 6.989, *p* < 0.010), and it had a significant negative influence (β = −0.127, *p* < 0.010), which indicated that extroverted personality negatively regulated the relationship between informativeness and arousal emotion, assuming that H4c was proved. In model 13, personalization and extroverted personality were added, and its explanatory power to the variance of arousal emotion had no obvious change, and it had no significant influence (β = −0.010, *p* > 0.050), which indicated that extroverted personality failed to regulate the relationship between personalization and arousal emotion, assuming that H4d was not proved.

Secondly, this study tested the role of extroverted personality in responsiveness, professionalism, informativeness and personalization in influencing pleasure emotion. Model 14 was only put into control variables, independent variables and adjustment variables, which accounted for 45.600% of the variance of pleasure emotion (*f* = 74.482, *p* < 0.001). In model 15, responsiveness and extroverted personality were further added, which had no obvious change in explanatory power to the variance of pleasure emotion and had no significant influence (β = −0.071, *p* > 0.050), indicating that extroverted personality could not regulate the relationship between responsiveness and pleasure emotion, assuming that H4e was not proved. In model 16, professionalism and extraversion personality were added, and its explanatory power to the variance of pleasure emotion was increased by 1.000% (*f* = 8.473, *p* < 0.010), and it had a significant negative influence (β = −0.121, *p* < 0.010), which indicated that extraversion personality negatively regulated the relationship between professionalism and pleasure emotion, assuming that H4f was proved. In model 17, informativeness and extroverted personality were added, and its explanatory power to pleasure emotion variance was increased by 1.600% (*f* = 13.252, *p* < 0.001), and it had a significant negative influence (β = −0.159, *p* < 0.001), which indicated that extroverted personality negatively regulated the relationship between informativeness and pleasure emotion, assuming that H4g was proved. In model 18, personalization and extroverted personality were added, and its explanatory power to the variance of pleasure emotion had no obvious change, and it had no significant influence (β = −0.035, *p* > 0.050), which indicated that extroverted personality failed to regulate the relationship between personalization and pleasure emotion, assuming that H4h was not proved. The results were shown in Table 5.

## Discussion

Based on the ABC attitude model and cognitive evaluation theory, this study explored the mechanism of interaction quality (responsiveness, professionalism, informativeness and personalization) between anchors and consumers on consumers’ emotion and impulsive purchase intention. We proposed 18 hypotheses, of which H4a, H4d, H4e, and H4h were not supported. The overall results of hypothesis testing in this study were shown in Table 6. In view of the hypothesis tests results, further discussion was as follows.

**TABLE 6:** Summary of hypothesis test results.

Number	Hypothesis	Results
H1a	Responsiveness has a significant positive effect on consumers’ arousal emotion	Proved
H1b	Professionalism has a significant positive effect on consumers’ arousal emotion	Proved
H1c	Informativeness has a significant positive effect on consumers’ arousal emotion	Proved
H1d	Personalization has a significant positive effect on consumers’ arousal emotion	Proved
H2a	Responsiveness has a significant positive effect on consumers’ pleasure emotion	Proved
H2b	Professionalism has a significant positive effect on consumers’ pleasure emotion	Proved
H2c	Informativeness has a significant positive effect on consumers’ pleasure emotion	Proved
H2d	Personalization has a significant positive effect on consumers’ pleasure emotion	Proved
H3a	Arousal emotion plays an intermediary role between interaction quality and consumers’ impulsive purchase intention	Proved
H3b	Pleasure emotion plays an intermediary role between interaction quality and consumers’ impulsive purchase intention	Proved
H4a	Extroverted personality negatively regulates the influence of responsiveness on arousal emotion	Not proved
H4b	Extroverted personality negatively regulates the influence of professionalism on arousal emotion	Proved
H4c	Extroverted personality negatively regulates the influence of informativeness on arousal emotion	Proved
H4d	Extroverted personality negatively regulates the influence of personalization on arousal emotion	Not proved
H4e	Extroverted personality negatively regulates the influence of responsiveness on pleasure emotion	Not proved
H4f	Extroverted personality negatively regulates the influence of professionalism on pleasure emotion	Proved
H4g	Extroverted personality negatively regulates the influence of informativeness on pleasure emotion	Proved
H4h	Extroverted personality negatively regulates the influence of personalization on pleasure emotion	Not proved

The findings that interaction quality between anchors and consumers (responsiveness, professionalism, informativeness, and personalization) had a significantly positive impact on consumers’ emotion (arousal and pleasure emotion). Based on cognitive evaluation theory, emotion does not originate from specific events or environment, but from people’s cognitive evaluation (Bagozzi et al., 1999). During the live streaming shopping, consumers attained a positive response in the interaction with anchors, resulting in a good experience, and further forming positive cognitive evaluation, resulting in arousal and pleasure emotion (Xu et al., 2021). The present study also finds consumers’ cognitive evaluation of external features will affect the generation of their emotion (Chang et al., 2012; Liu et al., 2020). That is, H1a to H2d were established. In addition, consumers’ emotion played a mediating role between interaction quality and impulsive purchase intention. Based on the ABC attitude model, consumers’ cognition will affect their emotion, and then affect their behaviors. The present study also finds that consumers’ perception of the atmosphere of online shopping will affect consumers’ arousal and pleasure emotion, which will lead to impulsive purchase (Koo and Ju, 2010; Liu et al., 2018). The results of this study also verified that in the live streaming shopping situation when consumers were stimulated by external factors, they would have cognitive evaluation, which would affect their emotion, and positive emotional reaction would prompt them to have impulsive purchase intention (Chang et al., 2012; Chan et al., 2017; Lee and Chen, 2021). That is, H3a and H3b were established.

Furthermore, the results showed that consumers’ extroverted personality significantly negatively moderated the relationship between professionalism, informativeness and consumers’ arousal and pleasure emotion. According to arousal theory, the arousal level of the cerebral cortex of extroverts is inherently low, and it is not easy to be activated or excited. They need to obtain more stimuli from the outside world to achieve a satisfactory level of arousal (Eysenck, 1994). Therefore, it was more difficult for anchors to meet consumers’ demand for stimulation in interaction. At the same level of stimulation, consumers with high level of extroverted personality would have less pleasure and arousal emotion. However, H4a, H4d, H4e, and H4h were not proved. The extroverted personality of consumers did not play a moderating role in the relationship between responsiveness, personalization, arousal and pleasure emotion of consumers. According to the two-factor theory, in the live streaming shopping situation, professionalism and informativeness of anchors in the interaction process were the basic abilities, which belonged to the hygiene factors for consumers, while the responsiveness and personalization belonged to the motivation factors (Herzberg, 1968). If anchors could respond in time and better meet the personalized needs of consumers, they would be more satisfied and generate positive emotion. Therefore, whether an extrovert or an introvert consumers were, the higher responsiveness, personalization they perceived, they would produce higher degree of arousal and pleasure emotion.

### Implications

Our current research confirmed the important influence of the interaction quality between anchors and consumers on consumers’ emotion and impulsive purchase intention, enriched the research content of live streaming shopping. It also expanded the research of interaction quality in online shopping situations, and laid the foundation for the in-depth research of live streaming shopping. This paper analyzed the mechanism of interaction quality on consumers’ emotion and impulsive purchase intention, opened the “black box” of impulsive purchase research. And this study laid the foundation for the in-depth study of consumers’ impulsive purchase intention in live streaming shopping. At the same time, this study also provided the following practical enlightenment for promoting the sustained and sound development of live streaming shopping.

First, for anchors and live broadcast merchants, while stimulating consumption by improving the quality of interaction with consumers, they should also pay attention to guiding consumers to maintain a certain rationality. First of all, anchors should respond quickly to the questions raised by consumers, and at the same time remind consumers not to spend blindly. Next, anchors should meet the personalized needs of consumers, provide targeted purchase suggestions, and help consumers better understand whether the products are suitable for them, so that consumers can select products in a targeted manner and avoid impulsive purchase. Then, anchors can make full use of their professional knowledge, provide consumers with more intuitive, three-dimensional and accurate product information, inform consumers of the specific use situation and target population of the product, and remind consumers who have no purchase demand or are not suitable for the product not to buy impulsively. Finally, anchors can also create a suitable communication atmosphere and adopt appropriate communication strategies, so as to better understand consumers’ needs and persuade consumers to avoid impulse when necessary. Therefore, merchants and anchors not only achieve the purpose of promoting brands and products, but also reduce the possibility of consumers’ impulsive purchase, thus reducing the return problem caused by impulsive purchase and further reducing the waste of enterprise resources.

Second, anchors must actively mobilize the emotion of consumers. During the live broadcast, anchors should actively interact with consumers to stimulate emotional fluctuations and make them feel happy.

Third, anchors should pay attention to the different personalities of consumers, so as to carry out targeted marketing strategies. Consumers’ personality traits affect their cognition, emotion and behavior. For those consumers with a high level of extroverted, they should be provided with more variety of stimulation, fun and novelty through different interaction methods and live content. For example, anchors can answer consumers’ questions and give professional explanations through bullet screen informativeness, mobilizing consumers’ enthusiasm for live broadcast interactive participation and the generation of positive emotion.

Finally, consumers should keep their senses and avoid impulsive consumption when shopping in daily live broadcasts. In the live broadcast room with high responsiveness, professionalism, informativeness and personalization, it is easy to make consumers’ emotion fluctuate, thus generating impulsive purchase intention. Especially when anchors make gimmicks through marketing means such as large coupons and limited purchase, consumers should calm down, carefully think about their actual economic ability and consumption demand, and avoid impulsive consumption and regret afterward.

### Limitations and Future Direction of Research

First, this study only studied the influence mechanism of the interaction quality between anchors and consumers on consumers’ impulsive purchase intention. However, the interaction in live streaming shopping includes consumers and anchors, consumers and other consumers, consumers and live broadcast platforms. In the future research, we can further study the influence mechanism of the interaction quality between consumers and other consumers, live broadcast platform on consumers’ impulsive purchase intention. Second, this study only studied consumers’ impulsive purchase intention. However, some of consumers’ purchasing behavior are purposeful purchase. In the further research, we can study the influence mechanism of the interaction quality on consumers’ purchase behavior. What’ more, we should further expand the sample size and source, and try to adopt the experimental method to improve the reliability of the conclusion.

## Conclusion

First, in the situation of live streaming shopping, the interaction quality between anchors and consumers will improve the emotional response of consumers. First of all, responsiveness, professionalism, informativeness and personalization will make consumers feel happy. In the context of live streaming shopping, anchors can interact with consumers in real time, create a pleasure atmosphere in the live broadcast room, exchange products information and problems, and make corresponding adjustments according to the needs of consumers. These aspects will make consumers feel happiness and value of live streaming shopping and enhance the shopping experience. This conclusion expands the application situation of interaction quality in offline service environment, proves its applicability in live streaming shopping environment, and makes up for the lack of empirical research on interaction quality in online shopping environment.

Second, consumers’ emotion, namely arousal and pleasure emotion, plays a partial intermediary role between interaction quality between anchors and consumers and consumers’ impulsive purchase intention. In the situation of live shopping, consumers have emotional reactions such as pleasure and excitement in the process of interacting with anchors, which leads to buy products impulsively.

Third, extroverted personality can regulate the relationship between the quality of interaction between anchors and consumers and consumers’ emotion, and extroverted personality plays a negative moderating role in the relationship between professionalism, informativeness and emotion. Under the same level of professionalism, informativeness stimulation, consumers with a high level of extroverted personality are more difficult to be awakened and less excited. This conclusion verifies the moderating effect of extroverted personality on the relationship between interaction quality and emotion, improves the deep-level influence mechanism of interaction quality on emotion, and provides some reference for the follow-up study on the moderating variables of the relationship between them.

## Data Availability Statement

The original contributions presented in the study are included in the article/supplementary material, further inquiries can be directed to the corresponding author/s.

## Ethics Statement

Ethical review and approval was not required for the study on human participants in accordance with the local legislation and institutional requirements. Written informed consent from the (patients/participants OR patients/participants legal guardian/next of kin) was not required to participate in this study in accordance with the national legislation and the institutional requirements.

## Author Contributions

YJ: concept proposal, original manuscript writing, analyzing and interpreting the data, and manuscript modify. GL: guidance and manuscript modify. LC: manuscript modify. All authors contributed to the article and approved the submitted version.

## Conflict of Interest

The authors declare that the research was conducted in the absence of any commercial or financial relationships that could be construed as a potential conflict of interest.

## Publisher’s Note

All claims expressed in this article are solely those of the authors and do not necessarily represent those of their affiliated organizations, or those of the publisher, the editors and the reviewers. Any product that may be evaluated in this article, or claim that may be made by its manufacturer, is not guaranteed or endorsed by the publisher.

## Footnotes

1 China Internet Network informativeness Center. The 49th Statistical Report on Internet Development in China [R]. Beijing: China Internet Network informativeness Center [CNNIC], 2022

2 Research Report on the Development of Online Live Broadcasting industry in China 2021 [R], 2021

3 China Consumers Association. Online Survey report on Consumer Satisfaction of Live streaming E-commerce Shopping [Z], 2020
